# Genome-Wide Association Study Identifies *HLA-DP* as a Susceptibility Gene for Pediatric Asthma in Asian Populations

**DOI:** 10.1371/journal.pgen.1002170

**Published:** 2011-07-21

**Authors:** Emiko Noguchi, Hiromi Sakamoto, Tomomitsu Hirota, Kaori Ochiai, Yoshimasa Imoto, Masafumi Sakashita, Fumitake Kurosaka, Akira Akasawa, Shigemi Yoshihara, Noriko Kanno, Yumi Yamada, Naoki Shimojo, Yoichi Kohno, Yoichi Suzuki, Mi-Jin Kang, Ji-Won Kwon, Soo-Jong Hong, Ken Inoue, Yu-ichi Goto, Fumio Yamashita, Takashi Asada, Hiroshi Hirose, Ikuo Saito, Shigeharu Fujieda, Nobuyuki Hizawa, Toru Sakamoto, Hironori Masuko, Yusuke Nakamura, Ichiro Nomura, Mayumi Tamari, Tadao Arinami, Teruhiko Yoshida, Hirohisa Saito, Kenji Matsumoto

**Affiliations:** 1 Department of Medical Genetics, Graduate School of Comprehensive Human Sciences, University of Tsukuba, Tsukuba, Japan; 2 Division of Genetics, National Cancer Center Research Institute, Tokyo, Japan; 3 Laboratory of Respiratory Diseases, RIKEN Center for Genomic Medicine, Yokohama, Japan; 4 Departments of Otorhinolaryngology Head and Neck Surgery, Faculty of Medical Sciences, University of Fukui, Fukui, Japan; 5 Kurosaka Pediatrics and Allergy Clinic, Himji, Japan; 6 Department of Allergy, Tokyo Metropolitan Children's Medical Center, Tokyo, Japan; 7 Department of Pediatrics, Dokkyo Medical University, Tochigi, Japan; 8 Department of Pediatrics, Graduate School of Medicine, Chiba University, Chiba, Japan; 9 Department of Public Health, Graduate School of Medicine, Chiba University, Chiba, Japan; 10 Childhood Asthma Atopy Center, Department of Pediatrics, Asan Medical Center, University of Ulsan College of Medicine, Seoul, Korea; 11 Department of Mental Retardation and Birth Defect Research, National Institute of Neuroscience, National Center of Neurology and Psychiatry, Tokyo, Japan; 12 Department of Psychiatry, Graduate School of Comprehensive Human Sciences, University of Tsukuba, Tsukuba, Japan; 13 Health Center, Keio University, Tokyo, Japan; 14 Department of Respiratory Medicine, Graduate School of Comprehensive Human Sciences, University of Tsukuba, Tsukuba, Japan; 15 Department of Allergy and Immunology, National Research Institute for Child Health and Development, Tokyo, Japan; University of Oxford, United Kingdom

## Abstract

Asthma is a complex phenotype influenced by genetic and environmental factors. We conducted a genome-wide association study (GWAS) with 938 Japanese pediatric asthma patients and 2,376 controls. Single-nucleotide polymorphisms (SNPs) showing strong associations (*P*<1×10^−8^) in GWAS were further genotyped in an independent Japanese samples (818 cases and 1,032 controls) and in Korean samples (835 cases and 421 controls). SNP rs987870, located between *HLA-DPA1* and *HLA-DPB1*, was consistently associated with pediatric asthma in 3 independent populations (*P*_combined_ = 2.3×10^−10^, odds ratio [OR] = 1.40). *HLA-DP* allele analysis showed that DPA1*0201 and DPB1*0901, which were in strong linkage disequilibrium, were strongly associated with pediatric asthma (DPA1*0201: *P* = 5.5×10^−10^, OR = 1.52, and DPB1*0901: *P* = 2.0×10^−7^, OR = 1.49). Our findings show that genetic variants in the *HLA-DP* locus are associated with the risk of pediatric asthma in Asian populations.

## Author Summary

Asthma is the most common chronic disorder in children, and asthma exacerbation is an important cause of childhood morbidity and hospitalization. Here, taking advantage of recent technological advances in human genetics, we performed a genome-wide association study and follow-up validation studies to identify genetic variants for asthma. By examining 6,428 Asians, we found rs987870 and HLA-DPA1*0201/DPB1*0901 were associated with pediatric asthma. The association signal was stretched in the region of *HLA-DPB2*, collagen, type XI, alpha 2 (*COL11A2*), and Retinoid X receptor beta (*RXRB*), but strong linkage disequilibrium in this region made it difficult to specifically identify causative variants. Interestingly, the SNP (or the HLA-DP allele) associated with pediatric asthma (Th-2 type immune diseases) in the present study confers protection against Th-1 type immune diseases, such as type 1 diabetes and rheumatoid arthritis. Therefore, the association results obtained in the present study could partially explain the inverse relationship between asthma and Th-1 type immune diseases and may lead to better understanding of Th-1/Th-2 immune diseases.

## Introduction

Asthma is the most common chronic disorder in children, and asthma exacerbation is an important cause of childhood morbidity and hospitalization. The prevalence of childhood asthma in Japan is 5.0% among school children in 2006 [1], and an estimated 300 million people worldwide have asthma [2]. Asthma is characterized by airway hyperresponsiveness and inflammation, tissue remodeling, and airflow obstruction. Infiltration of lymphocytes, mast cells, and eosinophils in the airways cause airway inflammation, and T helper (Th) type 2 cytokines play crucial roles in orchestrating the inflammatory responses; thus, asthma is considered a Th2-type immune disease.

Previously conducted genome-wide association studies (GWAS) for asthma identified association with the loci on chromosomes 17q21 (*ORMDL3* for Caucasian pediatric asthma, odds ratio (OR) = 1.45, *P* = 1×10^−10^) [3], 5q21 (*PDE4D* for pediatric asthma, OR = 0.6, *P* = 4.7×10^−7^) [4], 9q21.31 (*TLE4* for Hispanic pediatric asthma, OR = 0.6, *P* = 6.8×10^−7^) [5], and 1q31 (*DENND1B* for Europeans and African ancestries [6], OR = 0.77 and 1.41, respectively; combined *P* = 1.7×10^−13^). A GWAS for severe asthma identified association with the region between *RAD50* and *IL5* on chromosome 5q (OR = 1.64, *P* = 3.0×10^−7^) and *HLA-DR/DQ* (OR = 0.68, *P* = 9.6×10^−6^), but they did not include a replication dataset [7]. Recently, Moffatt *et al.* conducted a large-scale GWAS in Caucasian populations and identified 6 loci (*IL18R1*, *HLA-DQ*, *IL33*, *SMAD3*, *GSDMB/GSDMA*, and *IL2RB*) associated with asthma [8].

In the present study, we conducted the first GWAS in Asian population for pediatric asthma by using Illumina HumanHap550/610-Quad BeadChip (Illumina, San Diego, USA).

## Results

### GWAS analysis

**Figure 1:**
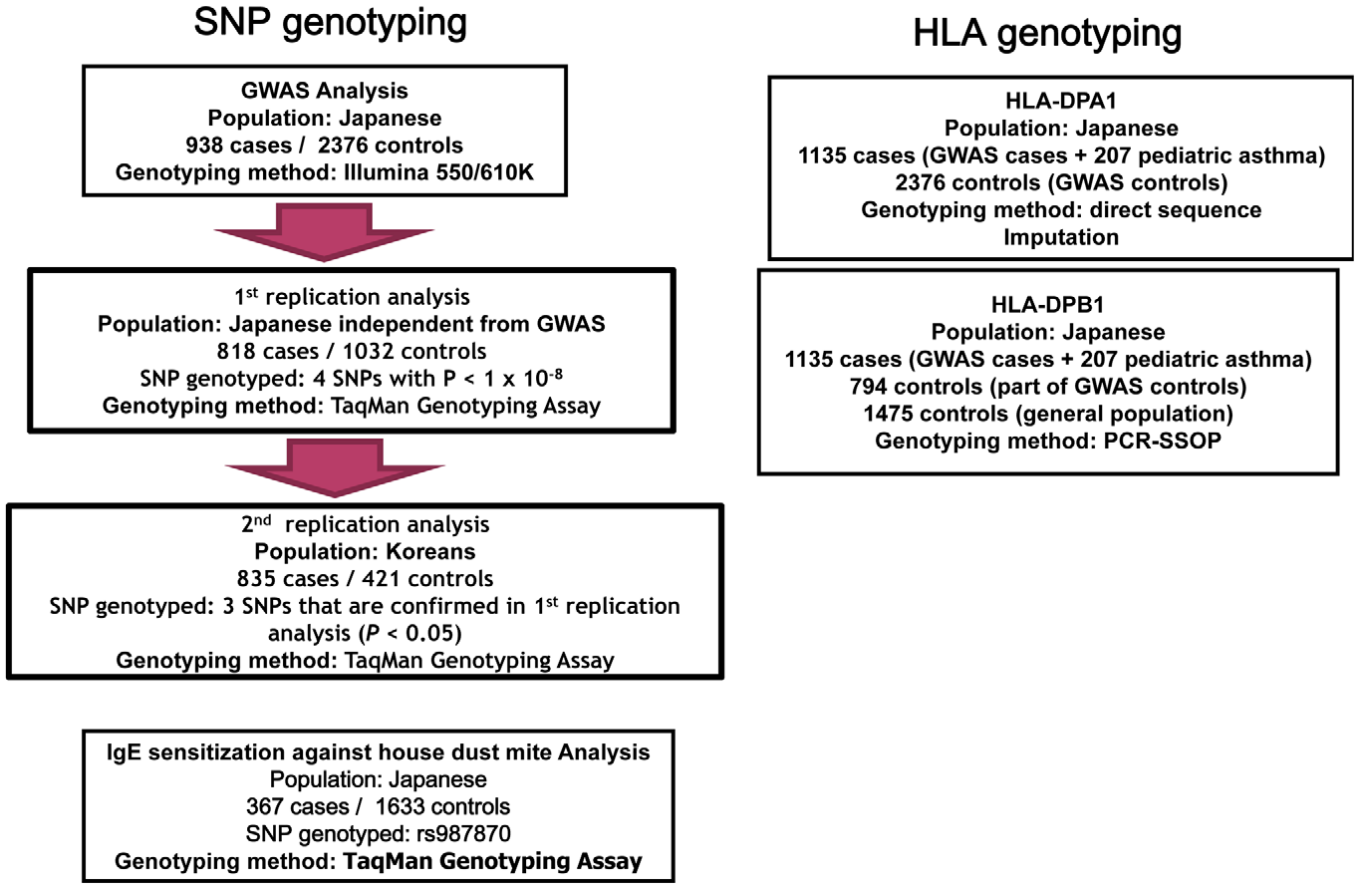
Flow chart of the present study.

**Figure 2:**
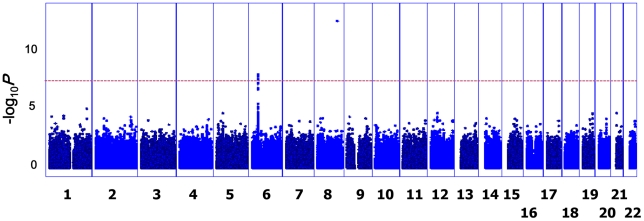
*P* values of GWAS. The Manhattan plot shows the Cochran–Armitage trend test *P* values for 938 cases of asthma and 2,376 controls; 450,326 autosomal SNPs were considered in the study. The dashed line indicates the genome-wide significance level (*P*<5×10−^8^).

**Figure 3:**
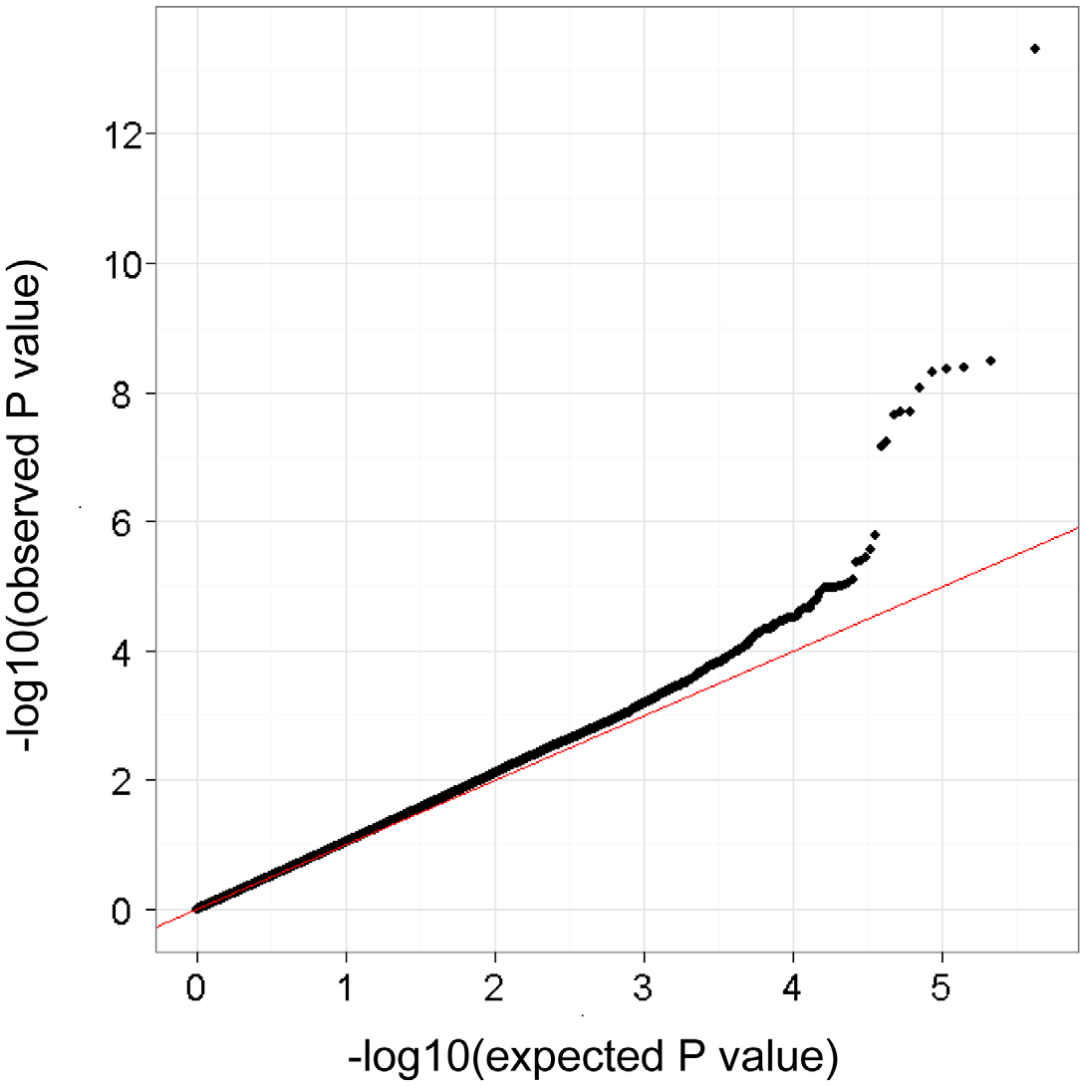
Quantile–quantile (Q–Q) plot of GWAS for pediatric asthma. The results of the Cochrane–Armitage trend *P* are plotted as dots and the line y = x is in red. The horizontal and vertical lines represent expected *P* values under null distribution and observed *P* values, respectively.

The GWAS flow chart is shown in Figure 1. We analyzed 450,326 SNPs in 938 cases and 2,376 controls, using standard quality control practices (Table S1). The genotypes in cases and controls were compared using the Cochran–Armitage trend test (Figure 2). There was only minor inflation of the genome-wide statistical results owing to population stratification (genomic control (λ_GC_) = 1.048; Figure 3). Five SNPs (rs3019885, rs987870, rs2281389, rs2064478, and rs3117230) showed strong association with pediatric asthma with *P*<1×10^−8^. Of these, rs2064478 and rs3117230 were in complete linkage disequilibrium (LD) (r^2^ = 1) with rs2281389. In order to validate the results of the GWAS, we tested the remaining 3 SNPs (rs3019885, rs987870, and rs2281389) in 2 independent replication cohorts comprising Asians (Japanese and Koreans), considering *P*<0.05 as significant replication.

Of these 3 SNPs, significant associations were noted at rs987870 in both cohorts (Table 1). To merge the findings of these studies, we conducted meta-analysis with a fixed-effects model by using the Mantel–Haenszel method. As shown in Table 1, the Mantel–Haenszel *P* value of 2.3×10^−10^ was noted for rs987870 (OR = 1.40, confidence interval (CI) = 1.26–1.55).

**Table 1:** Results of GWAS and replication studies for 4 SNPs.

SNP	Nearest	Allele^a^	Samples	MAF	MAF	OR (95%CI)^b^	*P* c	*P* e
	Gene			(asthma)	(control)			
rs3019885	SLC30A8	T/G	GWAS	0.41	0.31	1.55(1.39–1.73)	1.3×10^−14^	
			First replication (Japanese)	0.34	0.30	1.21(1.05–1.39)	8.7×10^−3^	
			Second replication (Koreans)	0.27	0.26	1.075(0.88–1.31)	4.7×10^−1^	
			Meta analysis (HM)^d^			1.34(1.24–1.45)	5.0×10^−13^	0.0011
rs987870	HLA-DPB1	T/C	GWAS	0.19	0.14	1.51(1.31–1.74)	7.5×10^−9^	
			First replication (Japanese)	0.17	0.14	1.26(1.05–1.50)	1.2×10^−2^	
			Second replication (Koreans)	0.12	0.10	1.34(1.01–1.76)	4.1×10^−2^	
			Meta analysis (HM)^d^			1.40(1.26–1.55)	2.3×10^−10^	0.33
rs2281389	HLA-DPB1	T/C	GWAS	0.23	0.17	1.47(1.29–1.68)	8.5×10^−9^	
			First replication (Japanese)	0.20	0.17	1.20(1.02–1.42)	2.9×10^−2^	
			Second replication (Koreans)	0.08	0.08	1.085(0.80–1.48)	6.1×10^−1^	
			Meta analysis (HM)^d^			1.33(1.20–1.47)	1.4×10^−8^	0.076

a The former allele represents the major allele.

b Odds ratio and 95% confidence interval (CI) of minor allele.

c *P* values of allelic model.

d Meta-analysis using Mantel-Haenszel approach.

e *P* values for heterogeneity test.

### HLA-DP association with pediatric asthma

**Figure 4:**
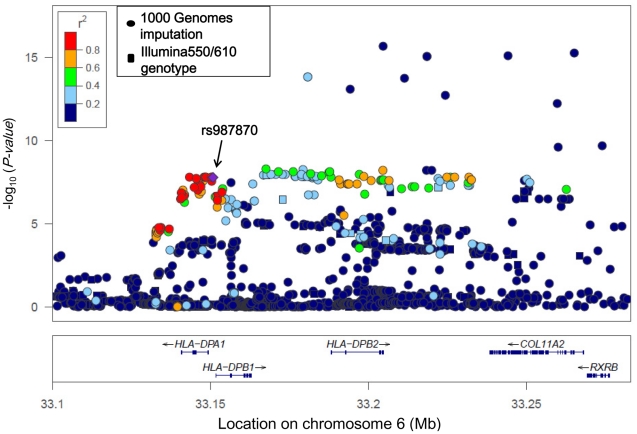
Association findings of genotyped (squares) and imputed (circles) SNPs in the *HLA-DP* region. SNP rs987870, which consistently showed an association with pediatric asthma in 3 independent populations, is located in the LD block between *HLA-DPA1* and *HLA-DPB1*. The color intensity of each symbol reflects the extent of LD with rs987870: from red (r^2^>0.8) to blue (r^2^<0.2). The physical positions are based on NCBI build 36 of the human genome.

The rs987870 is located between *HLA-DPA1* and *HLA-DPB1*. Genotype imputation using MACH [9] revealed association between asthma and the SNPs that were in strong LD with rs987870 (Figure 4, Table S2). Moreover, rs987870 C allele was in complete LD with DPA1*0201 (r^2^ = 1). We determined *HLA-DPA1* genotypes by using direct sequencing and MACH imputation of the data from 1135 cases and 2376 controls and found that DPA1*0201 was strongly associated with pediatric asthma (*P* = 5.2×10^−10^, OR = 1.52, Table 2). Then, we determined the *HLA-DPB1* genotypes in 1135 cases and 2296 controls and found that DPB1*0901 was associated with pediatric asthma (*P* = 2.0×10^−7^, OR = 1.49, Table 3). DPB1*0901 was in strong LD with DPA1*0201 and rs987870 C allele (*D* prime = 0.93). Because more than 90% of pediatric asthma patients were allergic to house dust mites, it is possible that the association was due to IgE reactivity (sensitization) against mites. We performed an association study for mite sensitization using independent adult subjects without allergic respiratory diseases such as asthma and perennial allergic rhinitis (367 subjects with house dust mite-specific IgE and 1633 subjects without mite-specific IgE). Subjects with house dust mite-specific IgE were non-allergic in terms of symptoms but possessed mite-specific IgE. Subjects without mite-specific IgE did not exhibit allergic symptoms. We did not find an association between rs987870 and mite sensitization (*P* = 0.54, OR = 1.07, Table S3).

**Table 2:** HLA-DPA1-rs987870 Haplotype analysis of pediatric asthma.

DPA1	rs987870	asthma	control	Odds ratio (95%CI)	*P* values
DPA1*0103	T	858(38%)	1866(39%)	0.98 (0.85–1.05)	0.29
DPA1*0201	C	439 (19%)	650 (14%)	1.52 (1.33–1.74)	5.5×10^−10^
DPA1*0202	T	957(42%)	2223(47%)	0.83 (0.75–0.92)	0.00046
DPA1*0401	T	3 (0.1%)	5 (0.1%)	1.26 (0.30–5.28)	0.75

**Table 3:** HLA-DPB1 allele frequency in pediatric asthma and controls.

Allele	Asthma	Control 1	Control 2	Asthma vs Control 1		Asthma vs Control 2		Asthma vs Control 1+2	
	n = 1135	n = 794	n = 1475	*P*	OR (95%CI)	*P*	OR (95%CI)	*P*	OR (95%CI)
DPB1*05:01	34.4%	36.5%	38.0%	0.18	0.91(0.80–1.04)	0.007	0.85(0.76–0.96)	0.013	0.87(0.79–0.97)
DPB1*02:01	22.4%	24.2%	24.3%	0.19	0.90(0.78–1.05)	0.11	0.89(0.79–1.02)	0.09	0.90(0.80–1.02)
DPB1*09:01	14.5%	10.1%	10.3%	5.5×10^−5^	1.51(1.23–1.84)	3.4×10^−6^	1.48(1.25–1.75)	2.0×10^−7^	1.49(1.28–1.74)
DPB1*04:02	10.0%	9.2%	9.6%	0.38	1.10(0.89–1.37)	0.57	1.05(0.88–1.27)	0.43	1.07(0.90–1.27)
DPB1*04:01	4.8%	7.1%	5.0%	0.0019	0.65(0.50–0.86)	0.64	0.94(0.73–1.21)	0.08	0.82(0.65–1.03)
DPB1*03:01	4.9%	4.7%	4.0%	0.76	1.05(0.78–1.41)	0.11	1.24(0.95–1.62)	0.21	1.17(0.92–1.48)
DPB1*02:02	3.7%	3.2%	3.4%	0.46	1.14(0.80–1.63)	0.61	1.08(0.80–1.45)	0.49	1.10(0.84–1.45)
DPB1*13:01	1.6%	1.5%	2.1%	0.85	1.05(0.62–1.77)	0.18	0.75(0.50–1.14)	0.38	0.84(0.57–1.24)
DPB1*14:01	1.8%	1.7%	1.4%	0.88	1.04(0.63–1.70)	0.258	1.28(0.83–2.01)	0.39	1.19(0.80–1.76)

## Discussion

Our GWAS in Asian populations found HLA-DP as susceptibility gene for pediatric asthma. Majority of pediatric asthmas are atopic (i.e., familial tendency to produce IgE antibodies against common environmental allergens) and possess specific IgE against the house dust mite. Mite sensitization is more prevalent in Asia than in Europe and is observed in 39% of the general adult population in Japan [10]. High prevalence of mite sensitization in asthmatic children has also been reported in Taiwan, where 94.2% of children with asthma are sensitized against *Dermatophagoides pteronyssinus* [11]. However, only a small subset of subjects with house dust allergy develop asthma [12].

We performed an independent association study for mite sensitization in adult subjects without allergic respiratory diseases and did not find an association between rs987870 and mite sensitization without symptoms. If the relative risk for mite sensitization in the individuals carrying a putative risk allele was 1.4 and the allele frequency was 0.15 compared to that in individuals without the allele, the statistical power of the sample size for mite sensitization study was 0.92 at an alpha level of 0.05. These results suggested that DPA1*0201 and DPB1*0901 may be associated with asthma rather than IgE production against house dust mite.

The association signal was stretched in the region of *HLA-DPB2*, collagen, type XI, alpha 2 (*COL11A2*), and Retinoid X receptor beta (*RXRB*) (Figure 4). Because of LD in this region, it is difficult to specifically identify causative variants. *HLA-DPB2* is a pseudogene. *COL11A2* encodes a component of type XI collagen called the pro-alpha2(XI) chain. Mutations in *COL11A2* have been associated with non-syndromic deafness, otospondylomegaepiphyseal dysplasia, Weissenbacher-Zweymüller syndrome, and Stickler syndrome (OMIM ID *120290). RXRB belongs to the RXR family and is involved in mediating the effects of retinoic acid. RXRB forms a heterodimer with the retinoic acid receptor and thus preferentially increases its DNA binding and transcriptional activity at promoters containing retinoic acid [13]. All SNPs showing strong association with asthma (*P*<1×10^−10^) were located in introns or intergenic regions. LD of these associated SNPs with rs987870 was not strong; therefore, it is likely that the functional effect is due to DPA1*0201 and DPB1*0901.

In HLA-DP, Caraballo *et al.* reported that DPB1*0401 is significantly decreased in patients with allergic asthma in Mulatto population (an admixture population of European and African ancestries) [14]. Apart from the study of Caraballo *et al.*, the association between *HLA-DP* alleles and asthma was restricted to occupational [15] or aspirin-induced asthma [16]. Howell *et al.* reported associations between HLA-DR genotype and asthma and between HLA-DPA1*0201 and IgE specific to grass pollen mix and the pollen allergen Phl p 5 [17]. Grass pollen allergy is not a major cause of asthma in Japan [18]; therefore, the HLA-DPA1*0201 association in the present study was less likely to be due to sensitization to grass pollen.

DPA1*0201 has also been reported to be positively associated with lower levels of rubella-induced antibodies [19], cytokine immune responses against measles vaccine [20], and ulcerative colitis [21], and negatively associated with type 1 diabetes [22]. DPB1*0901 was shown to be associated with systemic sclerosis [23], non-permissive mismatches for hematologic stem cell transplantation [24], ulcerative colitis [21], and Takayasu's arteritis [25]. *HLA-DP* molecules present short peptides of largely exogenous origin to CD4-positive helper T cells and other T cells, leading to subsequent immunological responses. T cells recognize complex formation between a specific HLA type and a particular antigen-derived epitope. Therefore, HLA molecules capable of binding a particular epitope can restrict T cell induced-immune responses, leading to association between particular HLA types and immune-related diseases. Type 1 diabetes is a Th-1 type immune disease. Varney *et al.* studied 1,771 type 1 diabetes multiplex families, analyzing them by the affected family-based control method [26], and found that DPA1*0201 has a protective effect on the development of type 1 diabetes (adjusted *P* = 5×10^−4^, OR 0.7) [22]. Epidemiologic studies have associated type 1 diabetes with lower prevalence of asthma and other allergic diseases [26], [27]. Also, the previous GWAS of rheumatoid arthritis, other Th-1 type immune disease, has shown that rs987870 C allele confers protection against rheumatoid arthritis [28]. These findings suggest that HLA-DPA1*0201 could determine Th1/Th2 dominance and could partially explain the inverse relationship between asthma and Th-1 type immune diseases.

Previous GWAS involving European, Mexican, and African populations showed association of asthma with SNPs located in several newly discovered genes. Our GWAS dataset supported an association between identical SNPs reported in *ORMDL3/GSDMB/GSDMA*, *IL5/RAD50/IL13*, *HLA-DR/DQ*, and *SMAD3* and pediatric asthma (*P*<0.05, Table S4). Two asthma GWA studies revealed an association of HLA-DQ with pediatric/adult asthma in Caucasians [7], [8]. HLA-DQ, like HLA-DP, is an αβ heterodimer of the MHC Class II type. Like HLA-DP, HLA-DQ recognizes and presents foreign antigens, but is also involved in recognizing common self-antigens and presenting those antigens to the immune system.

We failed to replicate the top SNPs of *PDE4D*, *TLE4*, *DENND1B*, *IL18R1*, and *IL2RB* that were reported in the original articles, but several SNPs in the regions surrounding *PDE4D* and *IL2RB* showed significant association when we set the significance level at *P* = 0.05 (Table S4). The different LD patterns/allele frequencies observed in *PDE4D* and *IL2RB* in Asians and Caucasians may explain the different SNP associations observed in different ethnic populations. rs1342326 in *IL33* was not polymorphic in the Asian population.

There were several limitations of the present GWAS. The controls for the GWAS and 1^st^ replication samples were from adult populations. Information regarding history of asthma in early childhood or other asthma-related information (i.e., status of allergic sensitization and lung function) was not collected for these controls. Therefore, we cannot exclude the possibility that our control samples may include subjects who outgrew asthma. The prevalence of pediatric asthma in Japan is around 5%; therefore, our GWAS samples have reduced power compared with that of selected controls. In the 1^st^ replication Japanese controls, subjects with present and past history of allergic diseases were excluded, and Korean controls in the 2^nd^ replication were non-allergic pediatric controls (Table S5).

The genomic control value in the present study was 1.053, indicating minor population stratification. The Japanese population comprises 2 clusters (Hondo and Ryukyu; Hondo is the mainland of Japan and Ryukyu is the name of the island south of Japan). We performed principal component analysis using EIGENSTRAT software [29] to identify subjects belonging to Ryukyu. Because 2nd or 3rd generation Chinese live in Japan, and the genetic population structure in Chinese differs from that in Japanese, we also performed principal component analysis to exclude Chinese subjects. Although hidden population stratification may exist, its influence on the final results is not expected to be significant.

rs3019885 is located in intron 2 of solute carrier family 30 (SLC30A8), and showed strong association in the GWAS population. The association was replicated in the independent Japanese samples, but not in the Korean population. SLC30A8 is a zinc efflux transporter expressed at high levels only in the pancreas; the GWAS revealed that variants of *SLC30A8* are associated with type 2 diabetes [30]. Japanese and Koreans are genetically close but we cannot exclude the possibility that the association of rs3019885 with pediatric asthma is population specific.

In conclusion, we performed the first GWAS in Asian population for pediatric asthma and found that DPA*0201/DPB1*0901 is strongly associated with pediatric asthma. The association with the HLA-DP locus emphasizes the importance of the HLA-class II molecules on the biological pathways involved in the etiology of pediatric asthma, and suggests that HLA-DP can be a therapeutic target for asthma.

## Materials and Methods

### Ethical statement

The study was approved by the institutional review board and the ethics committee of each institution. Written informed consent was obtained from each participant in accordance with institutional requirements and the Declaration of Helsinki Principles.

### Subject participants

Characteristics of pediatric asthma cases and controls are summarized in Table S5.

#### GWAS population

All subjects with asthma were child or child-onset (<15 years old) asthmatics in Japan. Patients were recruited from 3 pediatric hospitals and 1 pediatric clinic, and the diagnosis of the asthma in all patients was confirmed by specialists in pediatric allergology on the basis of the criteria of the National Institutes of Health, USA, with minor modifications.

The control cases for the GWAS were healthy Japanese adult subjects from Osaka (n = 964), Tokyo (n = 660), and Ibaraki (n = 778) who had no current history of asthma [31]. In the GWAS, we genotyped 978 cases with pediatric asthma and 2402 controls using Illumina HumanHap550v3/610-Quad Genotyping BeadChip (Illumina, San Diego, USA). Subjects from Osaka and Ibaraki were randomly selected from residents of Suita city and Tone town, respectively. Subjects from Tokyo were hospital workers from Keio University Hospital, Tokyo. We excluded samples considered duplicated, related (first- or second-degree relatives), or belonging to Han Chinese or Ryukyu. In total, 938 cases and 2376 controls were considered for further analysis.

#### First replication population (Japanese)

We recruited 818 subjects with childhood atopic asthma from the Osaka Prefectural Medical Center for Respiratory and Allergic Diseases, Dokkyo University School of Medicine, National Research Institute for Child Health & Development, National Sagamihara Hospital, and Chiba University Hospital. All subjects with bronchial asthma were diagnosed according to the criteria of the National Institutes of Health (National Heart, Lung, and Blood Institute, National Institutes of Health, 1991) by physicians who were asthma specialists [32], [33]. After the exclusion of individuals who had been diagnosed with asthma, atopic dermatitis, or nasal allergies by physicians' interviews, 825 healthy individuals were recruited from the Midousuji Rotary Club [32], [33]. Two hundred and seven control subjects who never had the symptoms of allergic rhinitis/asthma and did not show any sensitization to 7 common aeroallergens were recruited from Fukui [34].

#### Second replication population (Korean)

Patients with pediatric asthma were enrolled at Asan Medical Center, University of Ulsan College of Medicine, Seoul, Korea. The control subjects were age-matched children with no history of asthma or other allergic diseases, negative skin prick test, and normal total IgE values (<100 IU/mL) recruited from the same district (Seoul). Total of 835 cases and 421 controls participated in this study. The details of the patients and controls were described in a previous study [35].

#### Subjects for IgE sensitization against house dust mite

General populations for mite sensitivity study were recruited from Fukui [10] and Tsukuba in Japan. Total and specific IgE levels (produced in response to Japanese cedar, *Dermatophagoides*, *Dactylis glomerata*, *Ambrosia artemisiifolia*, *Candida albicans*, and *Aspergillus*) were measured using the CAP-RAST method (for Fukui samples; Pharmacia Diagnostics AB, Uppsala, Sweden) or MAST-26 (for Tsukuba samples; Hitachi Chemical Co. Ltd., Tokyo, Japan). Positive sensitization against house dust mite was defined as specific IgE levels against the house dust mite (*Dermatophagoides farinae* or *Dermatophagoides pteronyssinus*) greater than or equal to 0.70 IU/ml (class 2) or lumicount greater than 2.76 (class 2). Subjects with asthma (current or past) or perennial allergic rhinitis were excluded from the analysis. Sensitized subjects (Mite-positive) were non-allergic in terms of symptoms but possessed mite-specific IgE. Non-sensitized subjects (Mite-negative) did not show any allergic symptoms and did not have mite-specific IgE.

#### Subjects for *HLA-DPA1* typing

Cases with asthma included 938 subjects used in GWAS analysis and 207 Japanese subjects with child- or child-onset (<15 years) asthmatics recruited in Tsukuba. The diagnosis of asthma in all patients was confirmed by specialists in pediatric allergology on the basis of the criteria of the National Institutes of Health, USA, with minor modifications. The control subjects were 2378 subjects that were used in GWAS analysis. Because most of the DNA from the GWAS controls was not available for genotyping, and we found that imputation of the *HLA-DPA1* allele using GWAS results was highly accurate (error rate, 0.003), we decided to genotype the *HLA-DPA1* allele by direct sequencing and imputation. Among the subjects for *HLA-DPA1* genotyping (1135 cases and 2376 controls), genotyping of 383 subjects was performed by direct sequencing and genotyping of the remaining 3128 samples was performed by imputation.

#### Subjects for *HLA-DPB1* typing

Cases with asthma included 938 subjects used in GWAS analysis and 207 Japanese subjects with child- or child-onset (<15 years) asthmatics; the same as those used in *HLA-DPA1* typing. The control 1 subjects for *HLA-DPB1* typing were 794 healthy adult subjects from Tokyo and 399 subjects were the same as those in GWAS. The control 2 subjects (n = 1475) were general datasets from Japanese population samples publically available at http://www.hla.or.jp/hapro/top.html. Because most of the DNA from the GWAS controls was not available for genotyping, and the imputation of the DPB1 allele using the GWAS results was not possible, we used 794 healthy adult subjects from Tokyo and 399 subjects from the GWAS for *DPB1* genotyping (Control 1). The control 2 subjects (n = 1475) were general datasets from Japanese population samples publically available at http://www.hla.or.jp/hapro/top.html. The status of asthma or other allergic diseases for these samples is not available.

### Genotyping

Genotyping for GWAS was performed using the Illumina HumanHap550v3/610-Quad Genotyping BeadChip (Illumina), as per manufacturer's instruction.

In replication analyses, genotyping of each individual was performed with the TaqMan genotyping system (Applied Biosystems) on an ABI PRISM 7900HT Sequence Detection System (Applied Biosystems). PCR was performed on a 384-well format, and automatic allele calling was performed using ABI PRISM 7900HT data collection and analysis software, version 2.2.2 (Applied Biosystems).

HLA-DPB1 genotyping of 1135 cases, 794 controls (control 1) and 1475 controls (control 2) were performed with the WAKFlow HLA typing kit (Wakunaga, Hiroshima, Japan), as per manufacturer's instruction. First, the target DNA was amplified by polymerase chain reaction (PCR) with biotinylated primers specifically designed for each HLA-DPB1 locus. Then, the PCR product was denatured and hybridized to complementary oligonucleotide probes immobilized on fluorescent-coded microsphere beads. Concurrently, the biotinylated PCR product was labeled with phycoerythrin-conjugated streptavidin and immediately examined with the Luminex 100 system (Luminex, Austin, TX). Genotype determination and data analysis were performed with the WAKFlow typing software (Wakunaga).

*HLA-DPA1* genotyping was performed with direct sequencing of exon 2 with forward primer 5′-TCAGGATGCCCAGACTTTCAA-3′ and reverse primer 5′-CAGGGGGCACTTAGGCTTCC-3′, and with the sequencing primer 5′-TCAGGATGCCCAGACTTTCAA-3′ using the BigDye Terminator v.1.1 Cycle Sequencing Kit (Applied Biosystems) on an ABI PRISM 3130 Genetic Analyzer (Applied Biosystems).

### Statistical analysis

In the GWAS, we examined the potential genetic relatedness on the basis of pairwise identity by state for all of the successfully genotyped samples using the EIGENSTRAT software [29]. In the GWAS, we genotyped 978 cases with pediatric asthma and 2402 controls using Illumina HumanHap550v3/610-Quad Genotyping BeadChip (Illumina, San Diego, USA). Samples of duplicated (identical individual or monozygotic twin), first-, second-, and third-degree pairs were detected, and the individual with a lower call rate was excluded from further analysis. PCA was performed, and the results were combined with those obtained for our in-house Ryukyu and Han Chinese reference samples. Yamaguchi-Kabata *et al.* characterized the Japanese population structure using the genotypes for 140,387 SNPs in 7003 Japanese individuals, along with 60 European, 60 African, and 90 East-Asian individuals, in the HapMap project and found that the Japanese population is composed of 2 clusters (Hondo and Ryukyu) [36]. Hondo is the biggest island of Japan, and the island of Ryukyu is located in southern Japan. Also, we have 2nd or 3rd generation Chinese living in Japan, and Chinese present a different genetic population structure from Japanese. Therefore, we excluded samples belonging to Han Chinese or Ryukyu, and 938 cases and 2376 controls were considered for further analysis.

Cluster plots of SNPs were checked by visual inspection and SNPs with ambiguous calls were excluded. We excluded SNPs with a low genotyping rate (<90%), minor allele frequency less than 0.01 in either pediatric asthma cases or controls, or with Hardy-Weinberg equilibrium *P* value<10^−4^ in controls. Finally, 450,326 SNPs were used for the GWAS. Details regarding the exact number of remaining SNPs after applying each quality control criterion are available in Table S1.

The genomic control inflation factor (λ_GC_), defined as the median association test statistic across all SNPs divided by its expected value, was calculated by the method proposed by Devlin *et al.* [37]. GWAS and replication analyses were performed using the Cochran–Armitage trend test and χ^2^ test. The meta-analysis was performed with the Mantel–Haenszel approach as a fixed-effects model [38]. All statistical findings were reported without correction. The results of GWAS were plotted with GWAS GUI v0.0.2 [39]. HLA-DP region was plotted with LocusZoom [40]. The power calculation was performed with Genetic Power Calculator [41]. Quantile-quantile (Q-Q) plot was plotted with ggplot2 package [42] in R version 2.10.0 (http://www.r-project.org/).

Imputation of genotypes in the DP region was performed with MACH version 1.0 [9] with 1000 Genome Project data (1000G 2010-6 release, http://www.sph.umich.edu/csg/yli/mach/download/1000G-2010-06.html).

### HLA-DPA1 allele imputation

The HLA-DP region was in strong linkage disequilibrium and some DPB1 alleles were known to be linked with particular DPA1 alleles. First, we imputed HLA-DPA1 alleles by using the actual genotype data of samples obtained from Illumina HumanHap550v3/610-Quad (Illumina) and 1000 Genome Project data of Asian origin (JPT+CHB) (http://www.sph.umich.edu/csg/abecasis/MaCH/download/1000G-2010-06.html). The accuracy of the imputated data was confirmed by direct sequencing. The error rate of imputation was 1/352 (0.003).

## Supporting Information

Table S1 Number of remaining SNPs after applying each quality control criterion.
(XLS)

Table S2 SNPs that are strong linkage disequilibrium (r^2^>0.9) with rs987870.
(XLS)

Table S3 Association analysis for mite IgE sensitization.
(XLS)

Table S4 Genotyping data of the Japanese pediatric asthma GWAS.
(XLS)

Table S5 Characteristics of cases and controls.
(XLS)
